# Is sexual reproduction of high-mountain plants endangered by heat?

**DOI:** 10.1007/s00442-015-3247-0

**Published:** 2015-02-20

**Authors:** Ursula Ladinig, Manuel Pramsohler, Ines Bauer, Sonja Zimmermann, Gilbert Neuner, Johanna Wagner

**Affiliations:** Institute of Botany, Faculty of Biology, University of Innsbruck, Sternwartestraße 15, 6020 Innsbruck, Austria

**Keywords:** Alpine plants, Heat tolerance, Reproductive ecology, Risk assessment, Temperature stress

## Abstract

Strong solar irradiation in combination with still air and dry soil can cause prostrate high-mountain plants to heat up considerably and ultimately suffer heat damage. Such heat damage has been repeatedly shown for vegetative structures, but not for reproductive structures, which we expected to be particularly vulnerable to heat. Heat effects on cold-adapted plants may increase with rising global temperatures and the predicted increase in heat waves. We have tested the heat tolerance of reproductive versus vegetative shoots at different reproductive stages, comparing ten common plant species from different elevation belts in the European Alps. Plant samples were exposed to temperatures in 2-K steps of 30 min each between 42 and 56 °C. Heat damage was assessed by visual rating and vital staining. Reproductive shoots were on average 2.5 K less heat tolerant (LT_50_, i.e. the mean temperature causing 50 % heat damage, 47.2 °C) than vegetative shoots (mean LT_50_ 49.7 °C). Initial heat injuries (mean LT_10_) were observed at 43–45 °C in heat-susceptible species and at 45–47 °C in more heat-tolerant species, in at least one reproductive stage. Generally, heat tolerance was significantly higher during fruiting than during the bud stages and anthesis. Prostrate species with acaulescent buds and flowers tolerated heat better than those with caulescent buds and flowers. Petals were the most heat-susceptible plant structure and mature pollen the most heat tolerant. Based on these data, heat tolerance of reproductive structures appears to be adapted to the prevailing maximum temperatures which the plants experience during different reproductive stages in their environment. During hot spells, however, heat tolerance thresholds may be exceeded. More frequent heat waves would decrease the reproductive output and, consequently, the competitiveness of heat-susceptible species.

**Electronic supplementary material:**

The online version of this article (doi:10.1007/s00442-015-3247-0) contains supplementary material, which is available to authorized users.

## Introduction

High mountains are generally associated with a cold environment. However, due to strong solar irradiation, the surface temperatures of the short vegetation can considerably exceed free air temperatures (Cernusca 1976; Wilson et al. 1987). Conditions of still air and dry soil increase this effect (Körner and De Moraes 1979; Neuner et al. 1999; Buchner and Neuner 2003; Körner 2003). The extent of radiative warming essentially depends on the plant’s habit. Species with prostrate shoots, including rosette plants and cushion plants, may heat up to 30–40 K above air temperature, and erect plants up to 20 K (Salisbury and Spomer 1964; Körner and Cochrane 1983; Gauslaa 1984; Körner 2003; Larcher and Wagner 2009, 2010; Neuner and Buchner 2012). Different plant statures within a stand and a variable micro-topography lead to considerable differences in plant temperatures at a small scale (Scherrer and Körner 2010; Neuner and Buchner 2012).

Maximum leaf temperatures measured in full solar radiation during midsummer can reach, depending on the growth habit, between 30 and 50 °C (Gauslaa 1984; Körner and Larcher 1988; Buchner and Neuner 2003; Neuner and Buchner 2012), but have been recorded to reach up to 60 °C in individual cases (Larcher and Wagner 1976; Buchner and Neuner 2003). Plant temperatures that surpass 42 °C become critical and may exceed the heat tolerance threshold of that plant. Plants may respond to exposure to high temperatures with a short-term heat hardening to the rising temperature (Gauslaa 1984; Neuner et al. 2000; Buchner and Neuner 2003; Larcher et al. 2010; Neuner and Buchner 2012), but this response is not always sufficient for heat survival under extended summer conditions with strong irradiation and low precipitation (Neuner et al. 1999; Buchner and Neuner 2003; Ladinig and Wagner 2005). In heat-susceptible species, heat damage in leaves first occurs at around 43 °C (30-min short-term heating; Larcher and Wagner 1976), with heat damage increasing rapidly once the heat tolerance threshold is passed. In the majority of alpine species, 50 % heat damage (LT_50_) in vegetative aboveground organs occurs between 45 and 52 °C, and between 50 and 60 °C in the most heat tolerant species (data compiled for 79 species of the European Alps by Neuner and Buchner 2012). This high variability in heat tolerance can be found concomitantly among species with different growth habits in close proximity, as demonstrated by Buchner and Neuner (2001). The strong influence of habit is also expressed by the fact that mean heat tolerance in plants of the different mountain systems on earth is rather similar (Gauslaa 1984; reviewed in Körner 2003).

In contrast to vegetative organs, hardly any information is available on the heat tolerance of reproductive structures in high-mountain plants. Lowland plants are known to be most vulnerable to extreme temperatures during reproduction (Hedhly et al. 2003, 2008; Barnabás et al. 2008; Zinn et al. 2010; Hedhly 2011). Thus, it can be expected that actively growing reproductive structures of high-mountain plants are at greater risk of heat damage than vegetative ones. Evidence exists for *Sempervivum montanum*, whose inflorescences are fully damaged at 52 °C; the same temperature is sustained by vegetative rosettes without injury (Larcher et al. 2010). In a number of high Andean plants, heat tolerance of reproductive tissues ranged from 47–51.6 °C (LT_50_) which was 0.7–2.3 K lower than in leaves (Neuner and Buchner 2012).

In the study reported here, we compared the heat tolerance of whole reproductive shoots and individual reproductive structures to vegetative shoots in ten common plant species from different vegetation zones of the European Central Alps. The species selected for study differ in their growth form (dwarf shrubs, cushion plants, herbs) and habit (erect or prostrate with acaulescent and caulescent reproductive shoots). We addressed the following questions: (1) Are there differences in heat tolerance between vegetative and reproductive structures within a species? (2) Are there differences in heat susceptibility among the main reproductive stages (bud stage, flowering, fruiting)? (3) Is there a relationship between growth form, habit and elevational distribution and heat tolerance? (4) On the basis of in situ temperature maxima, what is the potential risk of suffering heat damage during reproductive development in the respective environment?

Based on our current state of knowledge on heat effects on plants, we expected that reproductive structures would be more susceptible to heat damage than vegetative ones. As heat tolerance is an adaptive trait within the predictable environmental temperature regime (Davis and Shaw 2001; Hedhly et al. 2008), we assumed that prostrate shoots with acaulescent reproductive shoots would tolerate heat better than erect growing ones and that species adapted to higher elevations would tolerate heat less well than species from the lower elevations.

This study has also to be seen in the context of rising global temperatures and the associated increase in the number of extreme weather events. The present study should reveal which of the study species might be particularly threatened when heat waves become more frequent and severe.

## Materials and methods

### Study species

The ten species investigated in this study and their characteristics are summarized in Table 1. Selection criteria for plant species were:

1. Species exhibit typical growth forms for the high-mountain environment (dwarf shrub, cushion plant, herb).
2. Species differ in their growth habit (vegetative shoots prostrate or erect, with acaulescent or caulescent reproductive shoots). The following habit types were distinguished: EAC, vegetative shoot erect, reproductive shoot acaulescent in bud stage *b*1, caulescent from bud stage *b*2 onward; PAC, vegetative shoot prostrate, reproductive shoot acaulescent in bud stage *b*1, caulescent from bud stage *b*2 onward; PAA, vegetative shoot prostrate, reproductive shoot acaulescent in all reproductive stages.
3. Species occur in different mountain vegetation zones: subalpine (i.e. the treeline ecotone), alpine, subnival (i.e. the alpine–nival ecotone) or nival (according to Ellenberg and Leuschner 2010).

All ten species occur commonly in and are typical of their respective vegetation zone.

**Table 1:** Characteristics of the ten high-mountain plant species included in the study

Growth form	Plant species	Abbrev.	Mountain belt^a^	Vertical distribution (m a.s.l.) in the Alps^b,d^	Sampling site^c^	Life form^d^	Type of reproductive shoot	Habit type^e^	Flowering time
Dwarf shrub	*Calluna vulgaris* (L.) Hull	*Cal vul*	Colline–alpine	0–2,700	P	Phanerophyte	Inflorescence	EAC	Late (July–September)
	*Loiseleuria procumbens* L.	*Loi pro*	Subalpine–alpine	1,800–2,800	P	Chamaephyte	Inflorescence	PAA	Early (May–June)
	*Rhododendron ferrugineum* L.	*Rho fer*	Subalpine–alpine	700–2,500 (3,000)	P	Phanerophyte	Inflorescence	EAC	Mid (June–July)
Cushion plant	*Saxifraga bryoides* L.	*Sax bry*	Subnival–nival	>2,000 m (4,200)	S	Chamaephyte	Solitary flower	PAC	Late (July–August)
	*Saxifraga caesia* L.	*Sax cae*	Alpine	1,600–2,600 (3,000)	H	Chamaephyte	Inflorescence	PAC	Late (July–August)
	*Saxifraga moschata* Wulfen	*Sax mos*	Alpine–nival	>1,600 (4,200)	H	Chamaephyte	Inflorescence	PAC	Mid (June–July)
	*Saxifraga oppositifolia* L.	*Sax opp*	Alpine–nival	1,600–3,800 (4,500)	H	Chamaephyte	Solitary flower	PAA	Early (May–June)
	*Silene acaulis* (L.) Jacq.	*Sil aca*	Alpine–nival	1,500–3,700	H	Chamaephyte	Solitary flower	PAA	Mid (June–July)
Perennial herb	*Cerastium uniflorum* Clairv.	*Cer uni*	Subnival–nival	2,000–3,500	S	Hemicryptophyte	Terminal flower	PAA	Late (July–August)
	*Ranunculus glacialis* L.	*Ran gla*	Subnival–nival	>2,000 (4,275)	S	Cryptophyte	Inflorescence	PAC	Early (June–July)

^a^Mountain belt in the European Alps: subalpine, treeline ecotone; subnival, alpine–nival ecotone; nival, ice-free areas within the glacier zone

^b^Vertical distribution according to Hegi (1975), Kaplan (1995), Landolt (1992), Zimmermann (1975), and Körner (2011); numbers in parenthesis give the highest localities recorded in the Swiss Alps

^c^Sampling site: H, Mt Hafelekar (2,350 m a.s.l.); P, Mt Patscherkofel (1,950 m a.s.l.); S, Stubai Glacier foreland (2,880 m a.s.l.)

^d^Life forms according to Raunkiaer (1934)

^e^Habit type: EAC, vegetative shoot erect, reproductive shoot acaulescent in bud stage *b*1, caulescent from bud stage *b*2 onward; PAC, vegetative shoot prostrate, reproductive shoot acaulescent in bud stage *b*1, caulescent from bud stage *b*2 onward; PAA, vegetative shoot prostrate, reproductive shoot acaulescent in all reproductive stages

### Sampling sites

*Calluna vulgaris*, *Loiseleuria procumbens* and *Rhododendron ferrugineum* were sampled in the subalpine dwarf-shrub belt (west-facing slope, 1,950–2,000 m a.s.l., Mt Patscherkofel 47°12′N, 11°27′E, Tyrolean Central Alps); *Saxifraga caesia*, *S. moschata*, *S.* *oppositifolia* and *Silene acaulis* were sampled in the alpine zone (west-facing sites, 2,300–2,350 m a.s.l., Mt Hafelekar, 47°18′N, 11°23′E, Northern Calcareous Alps); *Cerastium uniflorum*, *Ranunculus glacialis* and *Saxifraga bryoides* were sampled in the subnival zone (north-west-facing slopes of the glacier foreland of the Stubai Glacier, 2,800–2,880 m a.s.l., 46°59′N, 11°07′E, Tyrolean Central Alps). Plants were either excavated with root bales (cushion plants and herbs) or shoots were cut off (woody shrubs). Plant individuals were wrapped in moist filter paper and transported at temperatures between 10 and 15 °C in cooler bags to the laboratory within 1 h (treeline and alpine sites) and 2 h (subnival sites). All plants were collected in the morning when the diurnal heat tolerance was low (Buchner and Neuner 2003). Heat treatments took place immediately upon the arrival of the collected plants to the laboratory.

### Reproductive stages

During the 2009 and 2012 growing seasons, heat tolerance of aboveground vegetative and reproductive shoots was determined in the following reproductive stages: bud stages *b*1 (reproductive buds tightly closed; before peduncle/pedicel elongation in species of habit type EAC and PAC) and *b*2 (flower buds still closed but shortly before anthesis; during peduncle/pedicle elongation in EAC and PAC types); anthesis *a*; fruit stage *f* (early fruit development, seeds undergo histogenesis). In *C. vulgaris*, *L. procumbens*, *C. uniflorum* and *R. glacialis*, only the bud stage *b*2 was investigated. Depending on the state of reproductive development, the term “reproductive shoot” stands for a single flower bud, flower and fruit including the pedicel (*C. vulgaris*,* L. procumbens*,* R. ferrugineum*,* S. bryoides*, S*. oppositifolia*,* S. acaulis*,* C. uniflorum*) or the inflorescence bud, the inflorescence and the infructescence including the peduncle (*S. caesia*, *S. moschata*, *R. glacialis*). The term “vegetative shoot” refers to mature stems and leaves in *C. vulgaris*, *L. procumbens* and *R. ferrugineum*; leafy short-stem shoots in the saxifrages and *S. acaulis*; newly forming stems and leaves of the hemicryptophyte *C. uniflorum*; leaves of the cryptophyte *R. glacialis*.

### Heat treatments

Plant samples were exposed to temperatures in 2 K steps—ranging from the temperature causing 0 to 100 % heat damage—in hot water baths (Thermomix Braun; Melsungen, Germany). At each exposure temperature, 10–55 randomly selected reproductive shoots from at least ten individual plants were tested together with several leaves or, in the case of cushion plants, up to six short-stem vegetative shoots. The plant samples were loosely arranged on wet filter paper in heat-durable and watertight plastic bags. The bags were then plunged into the preheated water baths to bring them immediately to the exposure temperature. The exposure time was 30 min, as is standard in heat tolerance tests (Kreeb 1990).

### Assessment of heat damage

Heat-treated and reference samples (untreated control samples and fully heat-damaged samples that had been immersed in a 80 °C water bath) were embedded in moist cotton in small plastic boxes and kept in growth chambers (photoperiod 16/8 h, temperature range 15/5 °C; PGC-GL, Percival Scientific Inc., Perry, IA) for 3–4 days, which was the time required for tissue necrosis to develop in the case of injury. For vegetative shoots, the percentage of visually damaged areas was assessed. Reproductive shoots were first either rated as undamaged (all visible structures intact) or damaged (at least one structure damaged). The extent of visual damage was expressed as the percentage of damaged reproductive shoots per individual. In a second step, heat damage to single reproductive structures (pedicel, petals, stamens with immature pollen, style including stigma, ovary including ovules and placenta) was detected using the vital stain TTC (2,3,5-triphenyltetrazoliumchloride; Merck KGaA, Darmstadt, Germany). Living tissues and cells turn red due to the activity of dehydrogenases, which transform the colorless TTC into the red-colored triphenyl formazan. For each exposure temperature, we incubated ten flowers from ten randomly selected reproductive shoots from each of the heat-treated and control plants in a 0.5 % TTC solution in 5-ml glass vials following the measurement protocol of Neuner et al. (2013). To ensure a quick penetration of the TTC solution into the samples, ovaries were scarified with a razor blade and infiltrated with the TTC solution under vacuum. After 24 h of incubation in the dark at room temperature, the samples were stored in an 86 % glycerol solution (Rotipuran, Roth, Germany) until further analysis. The percentage of heat damage was assessed by comparing heat-treated with reference samples under a stereo microscope (Olympus SZH; Olympus Inc., Tokyo, Japan).

Viability data from each heat treatment temperature were randomly assigned to datasets (*n* = 10) and plotted against the treatment temperatures. For each dataset, we fit a classic logistic function using the software OriginPro 7G SR4 (OriginLab Corp., Northampton, MA). The following threshold values for heat damage were read from the curve-fitting protocol for each replicate: LT_10_, LT_50_, LT_90_ (temperatures causing 10, 50 and 90 % heat damage) and LT_100_ (lowest temperature causing 100 % heat damage). We calculated the mean LT_10_, LT_50_, LT_90_ and LT_100_ from the single values of each dataset.

### Investigation of heat tolerance of mature pollen grains

The heat tolerance of mature pollen was not unambiguously detectable by TTC and therefore was investigated separately via in vitro pollen germination and pollen-tube growth assays. Freshly opened anthers were collected in Eppendorf tubes and heated in a blockheater (Stuart Block Heaters, Camlab Inc., Cambridge, UK) for 30 min. Heat treatments were conducted at 5-K steps at temperatures from 40 °C until 100 % of the pollen grains had been heat-killed (no germination ascertainable). Pollen from ten different individuals, ten flowers each (*C. uniflorum*,* R. ferrugineum*,* S. caesia*,* S. moschata*,* S. bryoides*) and three to five flowers each (*R. glacialis*) were separately tested at each temperature step.

Heat-treated pollen of each individual was spread onto glass slides on solidified germination medium according to Boavida and McCormick (2007). Depending on the species, the optimum sucrose concentration was between 10 and 30 %. The glass slides were placed in moisture incubation chambers at 25 °C. Pollen germination counts were made at random in six fields per glass slide under a microscope at 20× magnification (Olympus, BX50) the day after. A pollen grain was classified as germinated if the length of the pollen tube was equal to or greater than the diameter of the pollen grain.

### Site temperatures

Air temperatures 2 m aboveground (referred to subsequently as 2 m air temperatures) from standard weather stations were provided by the Central Institute for Meteorology and Geodynamics, Austria (ZAMG) for Mt Patscherkofel (alpine zone, 2,246 m a.s.l., 47°12′31′′N, 11°27′38′′O) and Pitztal Gletscher (subnival zone, 2,840 m a.s.l., 46°55′36′′N, 10°52′46′′E, Tyrolean Central Alps). For the subalpine site (Mt Patscherkofel, 1,950 m a.s.l.) an automated weather station (CR10; Campbell Scientific, Logan, UT; operated by G. Wieser) provided the air temperature data.

Leaf canopy temperatures were recorded at hourly intervals at subnival sites (Stubai Glacier, 2,880 m a.s.l.; Pitztal Glacier, 2,840 m a.s.l.), alpine sites (Mt Hafelekar, 2,350 m a.s.l.) and a subalpine site (Mt Patscherkofel, 1,950 m a.s.l.) using small data loggers (Tidbit, Onset, Bourne, MA). We recorded measurements throughout the year at all sites. The study period differed in terms of duration for the sites: Mt Hafelekar, Stubai Glacier (2002–2012); Pitztal Glacier (2007–2009); Mt Patscherkofel (2009–2011). Temperature loggers were placed between short-stem shoots in plant cushions or mounted in the leaf canopy of *Rhododendron* shrubs. During the growing season, additional loggers were mounted on supports at the height of the flowers and shaded by white plastic caps to avoid overheating.

We also recorded the leaf temperatures of dwarf shrubs, cushion plants and herbs in their respective environment using fine-wire thermocouples connected to data loggers (CR10; Campbell Scientific) that collected temperature records from the sensors every 5 min and calculated 30-min means. The study periods were 1998–2004 and 2008 at subalpine sites, 1998–2000 and 2009 at alpine sites and 2009 at a subnival site. Bud and flower temperatures were repeatedly recorded during shorter periods.

Plant temperatures measured in individual leaves and flowers in the vicinity of the standard weather stations (distance from station 50–500 m) were screened for absolute temperature maxima. Using the recorded data on 2 m air temperature, we determined the mean number of summer days (June–August) with temperature maxima of ≥12 °C in the different temperature classes (range 2 K) for 2002–2012. To visualize heat accumulation in prostrate plants (habit types PAC in the *b*1 stage and PAA), daily maximum 30-min plant temperatures were related to the respective daily maximum 2 m air temperatures recorded on the same day at the same site.

### Infrared video thermography

Infrared thermography was carried out on *S*. *moschata* and *S*. *acaulis* at the alpine site on the west- and south-exposed slopes on Mt Hafelekar (2350 m a.s.l.) on 19 June 2012, a warm clear day, between 2 and 4 p.m. Maximum 2 m air temperature was 14.6 °C. The infrared camera (ThermaCAM S60; FLIR Systems AB, Danderyd, Sweden) was equipped with a close-up lens (LW 64/150) for macro images of single flowers. For field measurements, the camera was mounted on a tripod and connected to a notebook to control measurements and record the data. The infrared images were recorded at a measurement interval of 400 ms. Further analysis of the infrared images was carried out with the software ThermaCAM Researcher Pro 2.8 (Flir Systems AB).

Plant temperatures were also registered with a set of eight copper constantan thermocouples (Type T, solder junction diameter 0.3 mm) connected to a data logger (DaqPRO 5300; Fourier Systems, www.fouriersystems.com) at a measurement interval of 1 s to provide reference temperatures. The thermocouples were fixed to the different plant parts with a medical tape permeable to gases (3 M™ Transpore).

### Statistics

Heat tolerance data were normally distributed (checked by Q–Q Plots), allowing parametric tests. Significant differences in heat tolerance among species, species groups and reproductive stages, and between vegetative and reproductive shoots, were tested for using either the Student’s *t* test or one-way analysis of variance (ANOVA). In the case of homoscedasticity (checked by Levene’s test) the Bonferroni post hoc test was used, otherwise we used the Tukey post hoc test. The effects and interactions of the factors “species,” “reproductive stage” (bud stage *b*1, *b*2, anthesis, fruiting) and “reproductive structure” (pedicel, petals, style, ovary) on heat tolerance (LT_50_) were analyzed using a fixed effect GLM ANOVA. In all tests, the critical level of significance was *α* = 0.05. All statistical analyses were carried out using the statistical software SPSS (SPSS, Chicago, IL).

## Results

### Heat tolerance of vegetative shoots

**Fig. 1:**
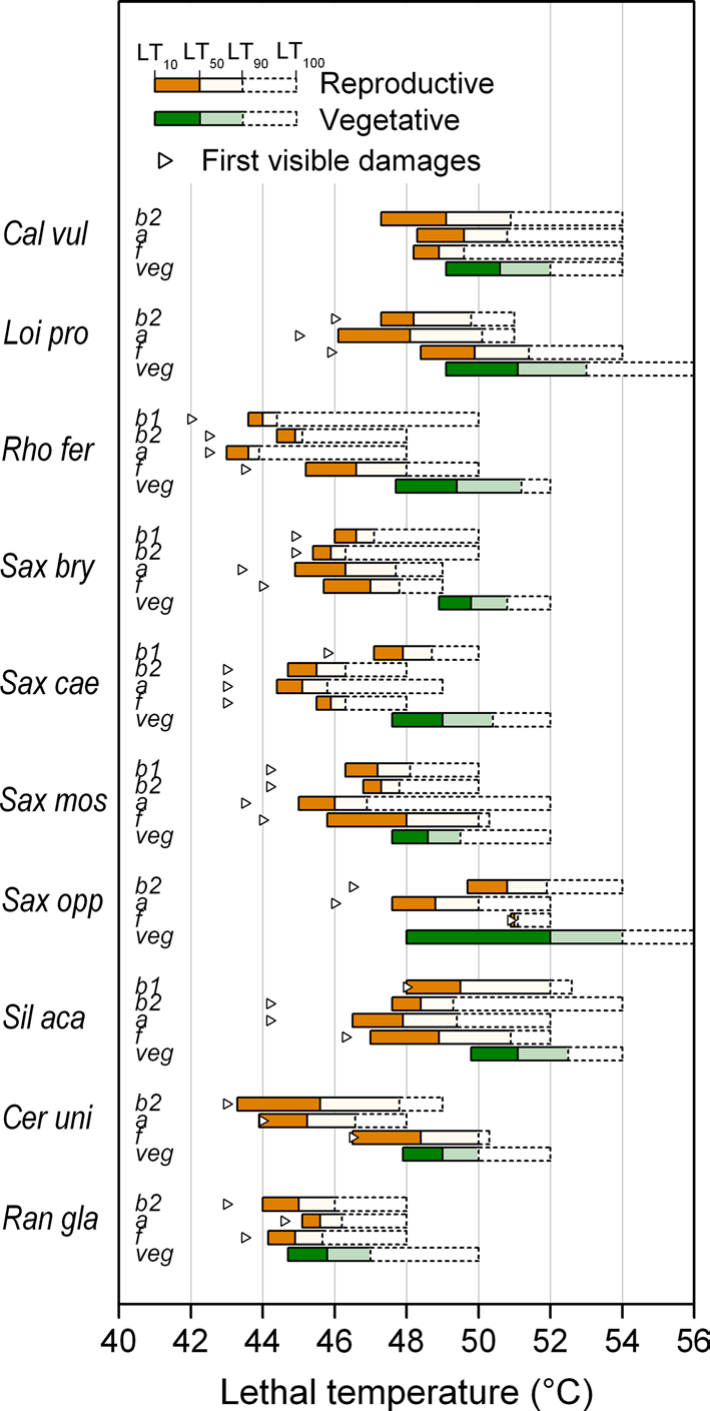
Heat tolerance of reproductive shoots in different reproductive stages (*b1* reproductive buds tightly closed; *b2* reproductive buds shortly before anthesis, during peduncle/pedicel elongation of caulescent shoots; *a* anthesis; *f* early fruit development) and of vegetative shoots (*veg*). *Horizontal bars* range from mean LT_10_ [lethal temperatures (LT) causing 10 % damage] to LT_100_ (LT causing 100 % damage) (from *left* to *right*). The range between mean LT_10_ and LT_50_ is given in *dark orange* (reproductive shoots) and *dark green* (vegetative shoots), the range between mean LT_50_ and LT_90_ is shown in *light orange* and *light green*, respectively. *Triangles* mark temperatures where the first damage became visible in single reproductive shoots. For the abbreviation of species names, see Table 1. For the definitions of LT_10_ to LT_100_, see section "Assessment of heat damage". In all investigated species, vegetative shoots were significantly more heat tolerant than reproductive shoots (intraspecies comparisons between LT_50_ values vegetative vs. reproductive across all developmental stages *P* < 0.001, *t* test). For statistical differences among reproductive stages, see Electronic SM Tables S1 and S2

Heat tolerance of the vegetative shoots varied significantly among species depending on the developmental stage (species × stage interaction, *F*_20,339_ = 5.74; *P* < 0.001). Mean heat tolerance of vegetative shoots (LT_50_) over all reproductive stages was highest in *S. oppositifolia* (>52 °C), followed by the dwarf shrubs *C. vulgaris* and *L. procumbens* and the cushion plant *S. acaulis* (around 51 °C). Heat tolerance was lowest in *R. glacialis* (around 46 °C). LT_50_ values for the remainder of species ranged between 48° and 50 °C [Fig. 1; Electronic Supplementary Material (ESM) Table S1 for mean LT_50_ values and statistics).

First heat damage (LT_10_) and 90 % damage (LT_90_) occurred at temperatures on average 1–2 K lower and higher, respectively, than LT_50_ (Fig. 1; ESM Table S2 for mean LT_10_ values and statistics). In half of the species, vegetative shoots were totally damaged (LT_100_) at about 52 °C, in *R. glacialis* already at 50 °C, and in *C. vulgaris*, *L.* *procumbens*, *S. oppositifolia* and *S. acaulis* at 54–56 °C.

**Fig. 2:**
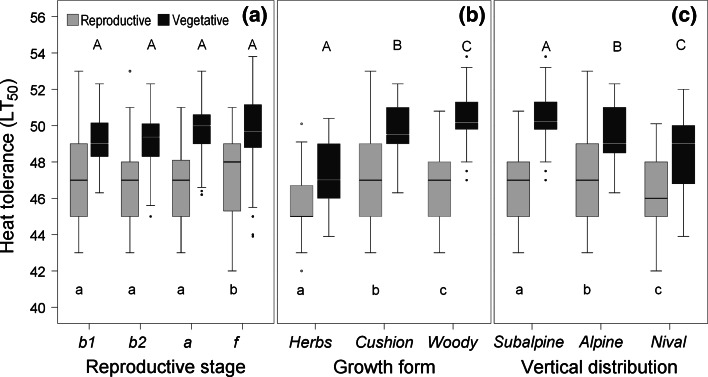
Heat tolerance (LT_50 _°C) of vegetative shoots (*black bars*) and of reproductive shoots (*gray bars*). Data for the species were pooled and grouped by different reproductive stages (**a**), different growth forms (**b**) and different mountain vegetation zones (**c**). *Box plots* show the median (*horizontal line inside box*), the 25th and 75th percentile (*top and bottom of box*), maximum and minimum values within the normal range (*whiskers*) and outliers (*circles*). *Different letters within subfigures* indicate statistical differences among different groups for vegetative shoots (*uppercase letters*) and for reproductive shoots (*lowercase letters*) by one-way ANOVA. Mean values of LT_50_ are significantly different between reproductive and vegetative shoots in all groups (*P* ≤ 0.001, *t* test)

Heat tolerance (LT_50_) of vegetative shoots tended to increase during reproductive development (Fig. 2a); however, this was only significant in* C. vulgaris*, *L. procumbens* and *R. ferrugineum* (for details see ESM Table S1). With respect to the growth form, woody species turned out to be most heat tolerant (mean ± standard deviation (SD): 50.4 ± 1.3 °C), followed by cushion plants (49.4 ± 1.4 °C) and herbs (47.3 ± 1.8 °C) (Fig. 2b; *P* < 0.001, one-way ANOVA). Species from higher elevations tolerated less heat than species from lower elevations (Fig. 2c; *P* < 0.001, one-way ANOVA).

### Heat tolerance of reproductive shoots

Reproductive shoots tolerated significantly less heat than vegetative shoots (Fig. 1), at least in single reproductive stages (for details see ESM Tables S1 and S2). Over all species and reproductive stages, the mean difference for LT_50_ amounted to 2.5 K ± 1.2 SD. The mean difference over all reproductive stages was small and not significant in *R. glacialis* (0.7 K ± 0.5), but it was clear-cut and significant in the remainder of species, particularly in *S. bryoides* (3.4 K ± 0.5) and *R. ferrugineum* (3.7 K ± 0.8). The difference in LT_50_ was larger in woody (2.6 K ± 1.2) and cushion plants (2.6 K ± 1.1) than in herbs (1.8 K ± 1.5).

Heat tolerance of reproductive shoots varied among species depending on the stage (species × stage interaction,* F*_21,1082_ = 5.13; *P* < 0.001). Reproductive shoots were particularly heat susceptible in *R. glacialis* throughout the whole reproductive developmental period (LT_50_ of approx. 45 °C), in *S. caesia* after peduncle elongation (LT_50_ 45–46 °C) and in *R.* *ferrugineum* from *b*1 until anthesis (LT_50_ of approx. 44 °C) (Fig. 1; ESM Table S1). Reproductive shoots of *S. oppositifolia* were the most heat tolerant (LT_50_ 49–51 °C), followed by *C. vulgaris* (LT_50_ of approx. 49 °C), *L. procumbens* and *S. acaulis* (LT_50_ of approx. 48–50 °C). First damage to single flowers occurred at considerably lower temperatures—at around 42–43 °C in* R. ferrugineum*, *C. uniflorum* and *R. glacialis*; at around 44 °C in *S. bryoides*, *S. caesia*, *S. moschata* and *S. acaulis,*; at >45 °C in *C. vulgaris*, *L. procumbens* and *S. oppositifolia*. This ranking of species according to heat susceptibility clearly shows that nival plants are among the most vulnerable of the plant species tested. The order of the remainder of species, however, does not reflect their environment (alpine, subalpine) but rather their habit (buds and flowers caulescent or acaulescent; for details see next paragraph).

Taking all species together, heat tolerance (TL_50_) was significantly higher during early fruiting than during the bud stages and anthesis (Fig. 2a; *P* < 0.004, one-way ANOVA). Pooling all reproductive stages together, reproductive shoots of herbs tolerated significantly less heat than those of cushion plants and woody plants (Fig. 2b; *P* < 0.001, one-way ANOVA). With regard to the elevational distribution, nival species were significantly more heat susceptible than species from the alpine and subalpine zone (Fig. 2c; *P* < 0.001, one-way ANOVA).

### Heat tolerance of individual reproductive structures

**Fig. 3:**
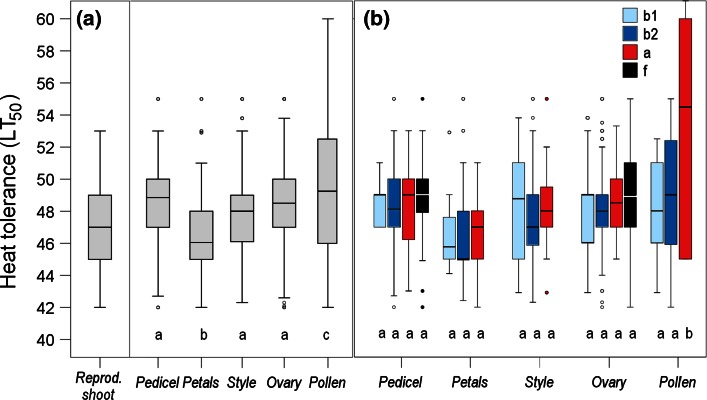
Heat tolerance (LT_50 _°C) of whole reproductive shoots and individual reproductive structures across all reproductive stages (**a**) and reproductive structures itemized by reproductive stages (**b**): *b*1 (*light blue*), *b*2 (*dark blue*), *a* (*red*), *f* (*black*). For the specification of the *box plots*, see caption to Fig. 2. *Different letters* in (**a**) indicate statistical differences among different reproductive structures and in (**b**) statistical differences among different reproductive stages within a reproductive structure (one-way ANOVA)

**Fig. 4:**
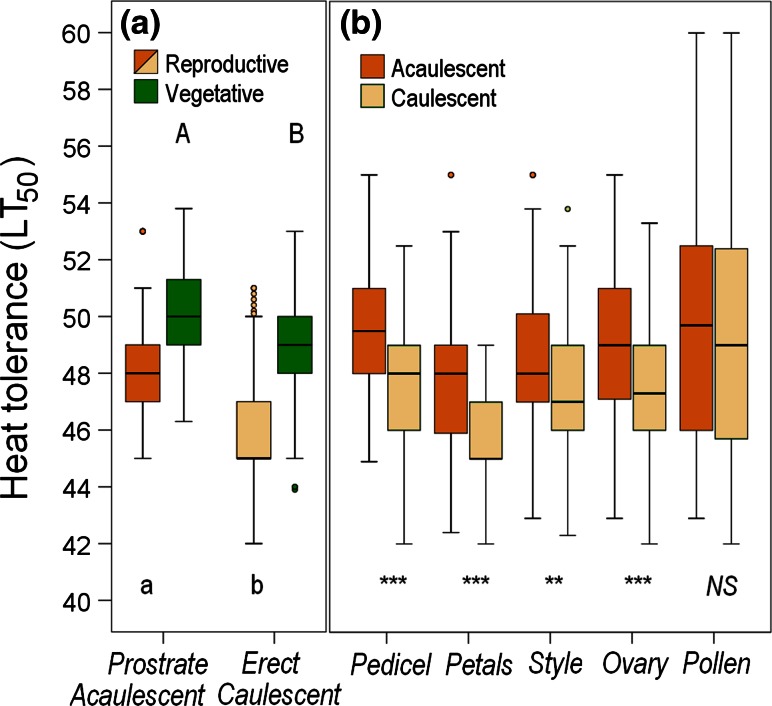
Heat tolerance (LT_50 _°C) according to shoot habit. **a** Prostrate vs. erect vegetative shoots (*green boxes*), and acaulescent vs. caulescent reproductive shoots (*orange boxes*), **b** individual reproductive structures of reproductive acaulescent (*dark-orange boxes*) and reproductive caulescent shoots (*light-orange boxes*). For the specification of the *box plots* see the caption to Fig. 2. *Different letters* in (**a**) indicate statistical differences between prostrate and erect vegetative shoots (*uppercase letters*, *P* < 0.001, *t* test) and between reproductive acaulescent and caulescent shoots (*lowercase letters*, *P* < 0.001, *t* test). *Asterisks* in (**b**) indicate significant differences between caulescent and acaulescent shoots within individual reproductive structures (**P ≤ 0.01,***P ≤ 0.001, *NS* not significant; *t* test)

Heat tolerance of individual reproductive structures was significantly species-dependent (species × structure interaction, *F*_26,1082_ = 3.33;* P* < 0.001). Across all species and reproductive stages, petals were significantly the most heat-susceptible plant structure, and pollen the most heat tolerant (Fig. 3a; *P* < 0.001, one-way ANOVA). When classified into reproductive stages, most structures tended to be most heat susceptible during the *b*2 stage, but the differences among stages within a structure were mostly not significant (Fig. 3b; stage × structure interaction, *F*_7,1082_ = 1.26; *P* = 0.269). Prostrate-growing vegetative shoots of the habit types PAC and PAA tolerated significantly more heat than erect vegetative shoots of the habit type EAC (Fig. 4a; *P* < 0.001, *t* test). Equally, acaulescent reproductive shoots (habit types PAA, PAC in bud stage *b*1) were significantly more heat tolerant (*P* < 0.001, *t* test) than caulescent reproductive shoots (habit type PAC after peduncle/pedicle elongation) and reproductive shoots of the EAC type in all stages. In detail, the differences were significant for all reproductive structures except for pollen (Fig. 4b).

**Fig. 5:**
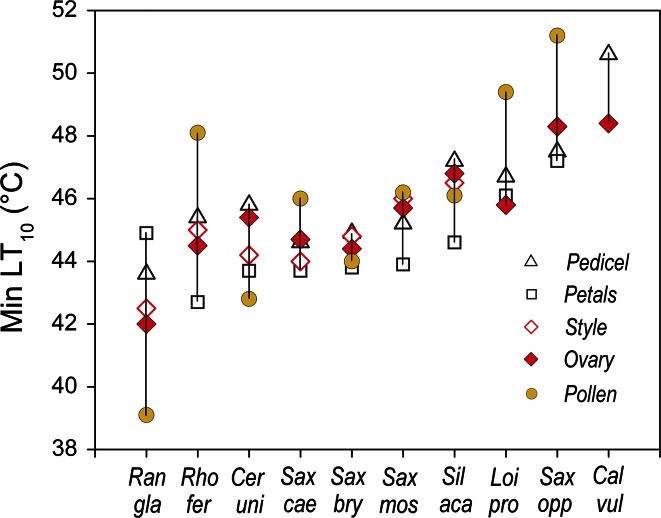
Lowest mean LT_10_ (°C) for the reproductive structures pedicel (*open triangle*), petals (*open square*), style + stigma (*red open diamond*), ovary (*red filled diamonds*) and pollen (*yellow filled circles*) observed across all reproductive stages within a species. *Vertical lines* range between the most heat-susceptible and most heat-tolerant reproductive structure. For the abbreviation of species names, see Table 1

Within a species at the same reproductive stage, the different reproductive structures showed—except for pollen—about the same threshold values for LT_50_ (for details see ESM Table S1). Significant differences in heat tolerance between the pedicel, style and ovary were found only in single species during single reproductive stages. The lowest mean LT_10_ value for each reproductive structure of each species observed across all reproductive stages was recorded for petals, whereas—apart from pollen—peduncles were the most heat tolerant structures (Fig. 5; see ESM Table S2 for species-specific details). The ranking of species according to the most heat-susceptible reproductive structure was found to be about the same as that for whole reproductive shoots (compare Fig. 1) and reflects the heat load that the respective plants may be subjected to at their natural growing sites. The nival species *R. glacialis* and *C. uniflorum* and the erect shrub *R. ferrugineum* were the most heat susceptible, cushion plants with caulescent flowers/inflorescences fell in the midfield range of heat susceptibility and flat-growing species of the PAA type and *Calluna* tolerated the most heat.

**Fig. 6:**
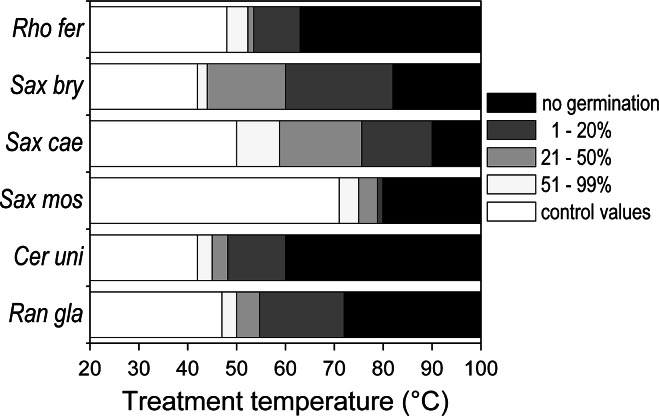
Heat tolerance of mature pollen grains. *Bars* pollen germination [as percentage of germination of respective control value (percentage germination of untreated pollen)] after heat treatment at different temperatures. *White bars* 100 % relative germination (compared to control value), *very light gray* 51–99 %, *light gray* 21–50 %, *dark gray* 1–20 %, *black* no germination. For the abbreviation of species names, see Table 1

As stated already, the heat tolerance of pollen differed considerably from that of the other reproductive structures. Pollen proved to be the most vulnerable structure during the bud stage in the nival species *R. glacialis* (LT_10_ 39 °C), and during anthesis in *C. uniflorum* and *S. bryoides* (Fig. 6; details in ESM Tables S1 and S2). In the remainder of the species, pollen tolerated higher temperatures than all other reproductive tissues; this tolerance was particularly pronounced in mature pollen (Fig. 6): pollen germination was largely unaffected until 50 °C; *S. moschata* pollen was unaffected up to 70 °C.

### Site temperatures and the risk of heat damage

**Fig. 7:**
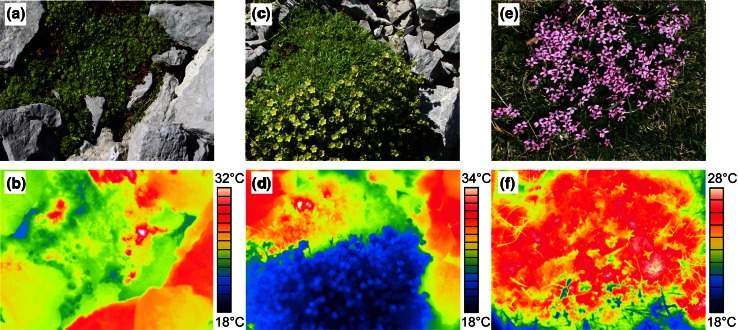
Temperature distribution within cushion plants measured with infrared thermography on a clear day at an alpine site. **a**,** b** *Saxifraga moschata* cushion with reproductive buds before peduncle elongation (**a**), with about the same temperature as the leaf canopy (**b**), **c**,** d** *S. moschata* cushion with caulescent inflorescences during anthesis (**c**), which are much cooler than the prostrate leaf canopy (**d**); **e**,** f** *Silene acaulis* cushion with acaulescent flowers (**e**) which have about the same temperature as the leaf canopy or are only slightly cooler (**f**)

**Fig. 8:**
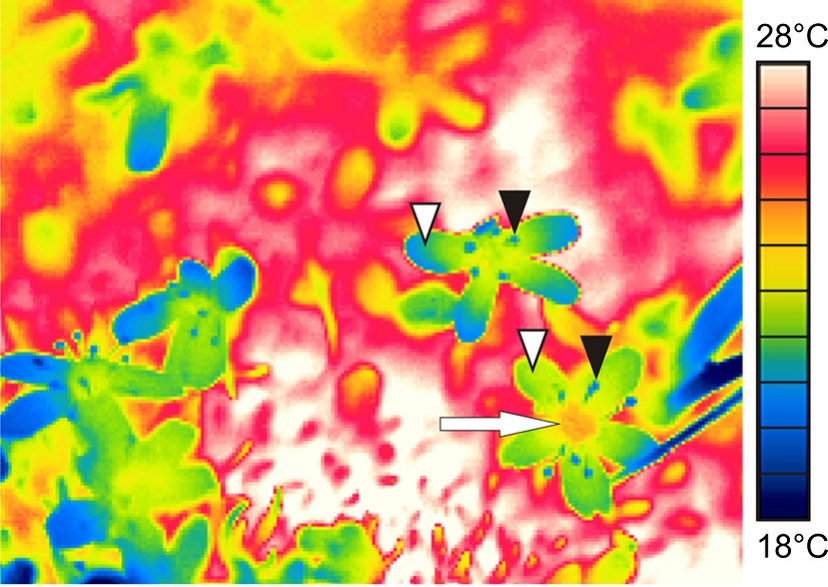
Temperature distribution in flowers of *Silene acaulis* displayed by infrared thermography. The ovary (*arrow*) heats up more than stamens (*filled arrowheads*) and petals (*open arrowheads*)

In situ infrared thermography on the cushion plant *S. moschata* on a bright summer day clearly showed that acaulescent reproductive buds had about the same temperature as the leaf canopy (Fig. 7a, b), whereas caulescent inflorescences were significantly cooler (Fig. 7c, d). In contrast, acaulescent flowers of *S. acaulis* were equally warm or only slightly cooler than the leaf canopy (Fig. 7e, f). Within a single flower, the ovary was the warmest structure, whereas stamens and petals heated up less (Fig. 8).

**Fig. 9:**
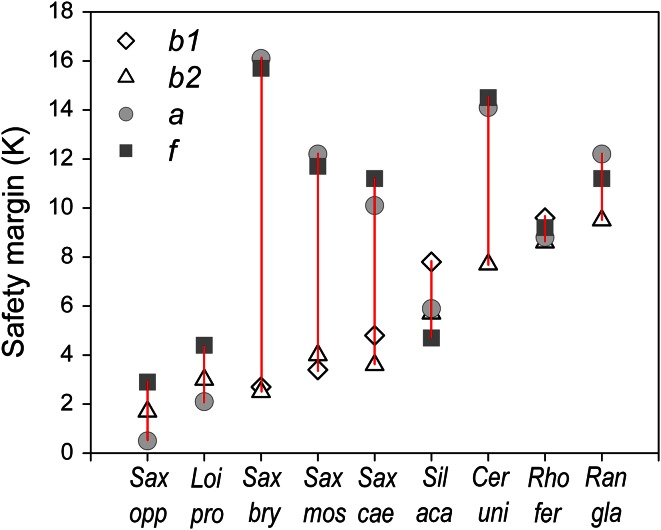
Safety margin (in Kelvin) between the temperature threshold causing LT_10_ and maximum temperatures of reproductive shoots recorded at the growing site during early bud stage *b*1 (*diamond*), late bud stage *b*2 (*triangle*), anthesis *a* (*gray circle*) and early fruit development *f* (*black square*) in the different species (for species names, see Table 1). *Vertical lines* mark the range between the lowest and highest safety margin during reproductive development

Absolute 2 m air temperature maxima between 2002 and 2012 in the summer months June–August were 25.9 °C at the treeline, 22.9 °C at the alpine site and 17.7 °C at the subnival site. Maximum plant temperatures were considerably higher and depended mainly on plant habit. Leaves of *R. ferrugineum* shrubs situated about 70 cm aboveground heated up to a maximum of 36 °C (30-min means), which did not pose any risk for vegetative shoots (lowest mean LT_10_ 45.4 °C) or reproductive structures at the same height (lowest mean LT_10_ 42.7 °C for petals) (Fig. 9). In contrast, the leaf canopy of the prostrate *L. procumbens* (PAA habit) reached 44 °C, which is near the temperature threshold causing first heat damage in reproductive tissues (lowest mean LT_10_ 45.7 °C for ovaries). Canopy temperatures measured in cushion plants usually remained <40 °C, and temperatures in flowers usually remained <35 °C. During hot and dry periods, however, canopy temperatures of cushion plants could reach up to 48 °C (*S. oppositifolia*, PAA habit), which is in the range of temperatures causing the first heat damage in acaulescent flower buds and flowers (compare Fig. 1). Leaves of the herbs *C. uniflorum* and *R.* *glacialis* reached maximum temperatures of 38 and 35 °C, respectively; their flowers and inflorescences, 30–33 °C. This results in a broad safety margin of about 8 K between maximum plant temperatures and LT_10_ for heat damage (Fig. 9) at the subnival growing sites.

**Fig. 10:**
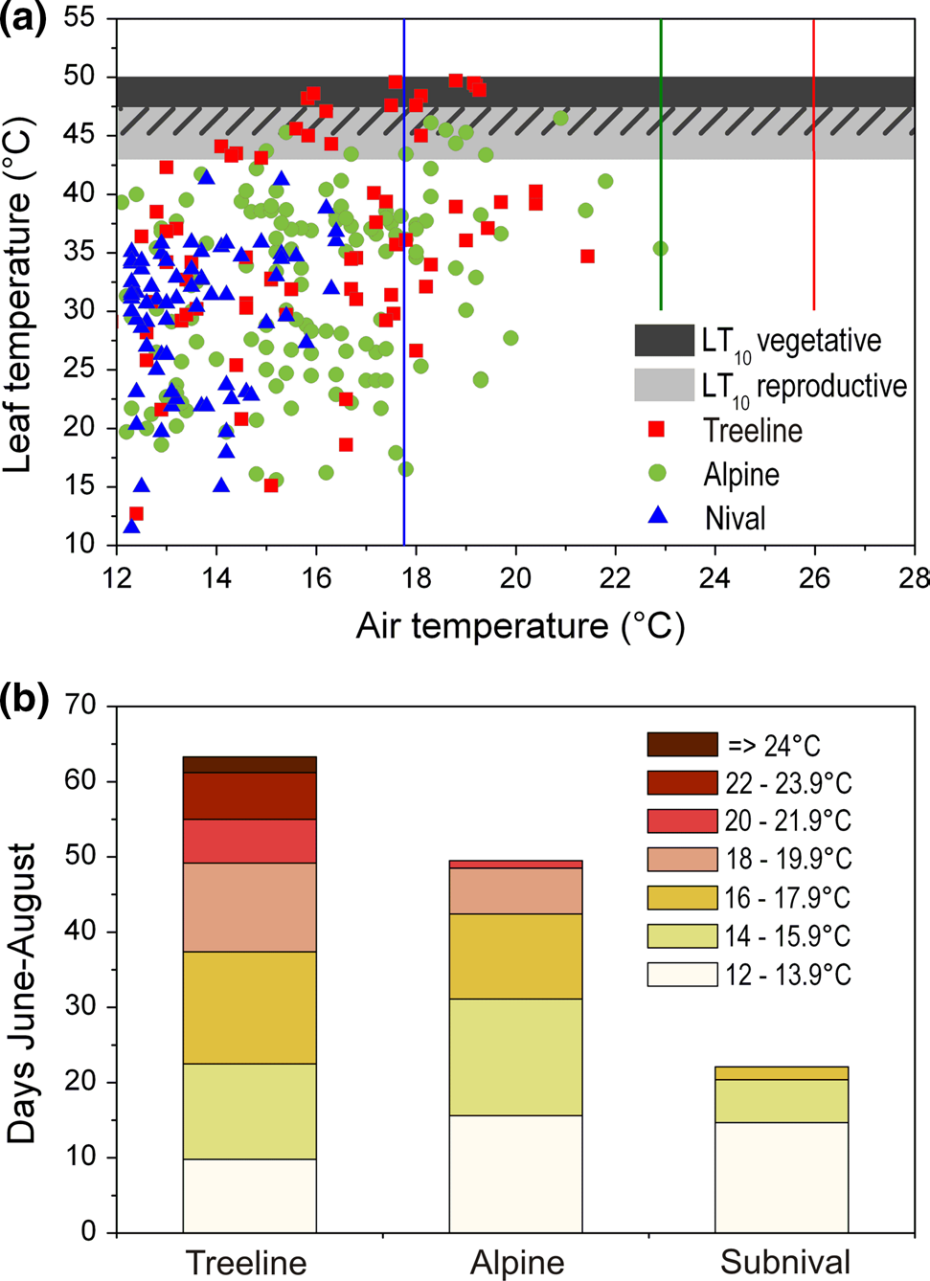
Risk of heat damage for species with prostrate reproductive and vegetative shoots in the different mountain zones. **a** 2 m air temperature maxima ≥12 °C and 30-min plant temperature maxima measured in single individuals on the same day at the treeline (*red squares*), at the alpine sites (*green filled circles*) and at the subnival site (*blue triangles*). *Horizontal bars* mark the range for LT_10_ in reproductive shoots (*gray*) and vegetative shoots (*black*). The ranges for reproductive high and vegetative low heat tolerance overlap (*hatched area*). *Vertical lines* mark maximum air temperatures occurring at the respective sites (*red* treeline,* green* alpine, *blue* subnival). **b** Mean number of summer days per season (June–August) and site with 2 m air temperature maxima in the specified temperature classes

At all sites, plant temperatures of >40 °C were recorded nearly exclusively on days on which the 2 m air temperature maxima were ≥12 °C. On days exceeding this air temperature threshold, the canopy of prostrate plants was on average 19.0 K ± 10.1 SD (maximum 33 K) warmer than the air temperatures at the treeline site, at the alpine site 16.1 K ± 7.4 (maximum 30 K), and at the subnival site 15.0 K ± 8.0 (maximum 27.5 K). When the maximum canopy temperatures are plotted against the maximum 2 m air temperatures on the same days and combined with temperature thresholds for LT_10_ in reproductive and vegetative shoots (Fig. 10a), there is practically no risk of heat damage at subnival sites, but there is a potential risk at the alpine and subalpine sites. Based on this plot, for heat-susceptible species, daily maximum air temperatures of >14 °C may already pose a risk to reproductive tissues; for more heat-tolerant species, the risk range would begin at 16 °C. For vegetative shoots, the critical threshold air temperature is 16 °C for heat-susceptible species and 18 °C for more heat-tolerant species. On the basis of the 2 m air temperatures, the mean number of risk days per summer season on which prostrate plants could potentially suffer from heat damage is then rather high at the treeline (53.5 ± 5.5 days, range 32–82 days), where 25.9 ± 6.5 days be classified as high-risk days (air temperatures of >16 °C) (Fig. 10b). In the alpine zone, there are 33.8 ± 5.6 risk days and 18.4 ± 4.7 high-risk days.

## Discussion

### Comparison of heat tolerance in vegetative versus reproductive shoots

Among the ten study plants, heat tolerance of vegetative shoots was in the range of earlier findings (Buchner and Neuner 2003; compiled in Neuner and Buchner 2012): mean LT_50_ for herbs was around 46 °C for *R. glacialis* (45–48 °C) and 49 °C for *C.* *uniflorum* (47 °C); for cushion plants it was between 48 and 52 °C (46–55 °C) and for dwarf shrubs between 48 and 51 °C (46–52 °C), where the values in parenthesis are LT_50_ values for the same species or growth form found in earlier studies. The variability among different investigations for the same species might result from different environmental preconditioning at different investigation sites, including daytime and short-term heat hardening during warmer periods (Gauslaa 1984; Neuner et al. 2000; Buchner and Neuner 2003; Larcher et al. 2010; Neuner and Buchner 2012), but also from different experimental methods (Buchner et al. 2013).

As expected, reproductive structures tolerated less heat (2.5 K on average) than vegetative ones; heat susceptibility tended to be highest during peduncle/pedicel elongation and/or anthesis, likely due to expanding and differentiating cells being most vulnerable to stress due to complex structural changes. Therefore, these phases generally become a weak link under temperature stress (Taschler et al. 2004; Neuner and Beikircher 2010; Zinn et al. 2010; Ladinig et al. 2013). In most species, the petals became heat damaged before other reproductive structures showed injuries. In addition to the vulnerability of petal tissues, heat stress-induced ethylene or abscisic acid synthesis might have accelerated the decay (Tripathi and Tuteja 2007; van Doorn and Woltering 2008). Except for *R. glacialis* and *L. procumbens*, pistils (stigma, style and ovary together) tolerated up to 3 K (*S. moschata*, *S. bryoides*) higher temperatures than petals, at least during one reproductive stage. In particular, the compact pistils of the saxifrages sustained the most heat, possibly related to the center of a flower heating up most due to low convection and irradiation potentially being trapped inside the bowl-shaped flowers (compare Fig. 8; Kevan 1975; Stanton and Galen 1989; Luzar and Gottsberger 2001; Galen and Stanton 2003; Rejšková et al. 2010; Dietrich and Körner 2014).

Among all reproductive structures, pollen showed highest variability in heat tolerance. Pre-anthesis pollen was the weakest link in the heat-susceptible species *R. glacialis* and *C. uniflorum*, but was the most heat-tolerant structure in most of the remaining species. Mature pollen was mostly affected at temperatures of ≥50 °C. Full loss of germinability, however, was caused by temperatures (60–90 °C) which are not to be expected at the natural growing sites. Heat susceptibility of mature pollen generally depends on its water content at dispersal, which can range from a small percentage up to about 70 % (Nepi et al. 2001; Franchi et al. 2002). Dry pollen is desiccation tolerant and because of high levels of late embryogenesis abundant (LEA) proteins better resists temperature extremes, whereas hydrated pollen does not (Franchi et al. 2011; Firon et al. 2012). Although the pollen water content of the study species is not known, its typical properties (Franchi et al. 2002) suggest that pollen of saxifrages and of *R. glacialis* is desiccation tolerant and that of *C. uniflorum* and *R. ferrugineum* is desiccation-susceptible.

Overall, it is to be expected that single reproductive structures of heat-susceptible species are already heat injured between 42 and 43 °C and that those of more heat-tolerant species become damaged at temperatures of 44–46 °C onwards. Depending on the structure concerned, even localized and slight heat damage can result in a total functional loss of a reproductive unit. Damage in the pedicel or peduncle automatically leads to the dieback of the whole flower or inflorescence (Neuner et al. 2013). Heat injury to the stigma and style leads to a complete loss of the progeny. Impairment of the corolla reduces the attractiveness for pollinators, which possibly leads to a reduced reproductive fitness of a flower, but not necessarily to a total reproductive loss. Damage to pollen affects the male fitness of the individuals concerned. Depending on the number of affected individuals within a population the genotypic variability of the progeny would become more or less reduced.

The expectation that reproductive structures in nival species are more susceptible to heat than those of species from lower elevations was only partly confirmed by our results. The nival species *R. glacialis* was clearly the most heat susceptible of the study plants, with little difference between reproductive and vegetative structures. This is consistent with earlier findings on leaf functions showing impairment from about 40 °C onward (Larcher et al. 1997). Reproductive structures of the nival species *S. bryoides* and *C. uniflorum* tolerated slightly more heat. The reproductive structures of the subalpine *R.* *ferrugineum* and alpine *S. caesia* had about the same low heat tolerance, which is in contrast to the relatively higher heat tolerance shown by their vegetative shoots. *C. vulgaris* (subalpine-alpine), *L. procumbens* (subalpine-alpine), *S. oppositifolia* and *S. acaulis* (both alpine-nival) tolerated the most heat. This ranking clearly shows that heat tolerance of reproductive structures, similar to that of vegetative organs, primarily depends on the species-specific heat load prevailing in the respective environment. Species clearly have a basic heat tolerance according to the heat load they usually experience. Starting from this basis, heat tolerance can, at least in leaves, increase in response to rising temperatures (Gauslaa 1984; Neuner et al. 2000; Buchner and Neuner 2003; reviewed in Neuner and Buchner 2012). We do not know whether reproductive structures are capable of heat hardening. It is however assumed that heat hardening is—similar to frost hardening (Neuner et al. 2013)—limited in the permanently structurally and functionally changing reproductive tissues.

### Risk assessment for heat damage of reproductive shoots

Daily air temperature maxima of >14 °C—temperatures at which prostrate plants can potentially heat above the temperature threshold causing heat damage—occurred on 60 % of summer days at the treeline and on 30 % of days in the alpine zone. However, as shown in Fig. 10a, measured plant temperatures in the risk range remained mostly below the damage threshold, indicating that high air temperatures alone do not necessarily cause lethal overheating in plants. As long as plants are sufficiently supplied with water and transpirational cooling is effective, the risk of lethal overheating may be low (Körner and De Moraes 1979; Neuner et al. 1999). However, the risk of overheating is high under conditions of high irradiation, calm air and dry soils, as shown by Neuner et al. (1999) and Buchner and Neuner (2003). Principally, the risk potential for individual plants differs according to the microsite conditions and the resulting small-scale temperature mosaic (Scherrer and Körner 2010). In cases of longer heat waves combined with drought, however, extensive heat damage at the community level is to be expected, as observed in the exceptionally warm summer 2003 (see following text).

Critical overwarming of plants can occur at any time during the summer months June–August and less often in May. Therefore, heat-endangered species are prone to suffer from heat damage during all reproductive stages. Daily temperature maxima are usually measured at midday and during the early afternoon. In our study, threshold values refer to 30-min short-term heating. As heat effects are dose-dependent (Kappen and Zeidler 1977), temperature thresholds for heat damage may become lower in cases of extended heating. In general, the dose effect is poorly investigated and should be considered in future studies on heat effects in plants.

We were able to show that acaulescent buds, flowers and fruits near the ground have the highest potential to suffer damage from heat. This is particularly true for the flat-growing dwarf shrub *L. procumbens* and the cushion plant *S.* *oppositifolia*, which at their respective growing sites reach maximum temperatures in the range of the heat damage threshold (30-min basis). When exposed to prolonged heating, the buds of the cushion plants* S. caesia*, *S. moschata* and *S. bryoides* (at lower sites) might be endangered. The remainder of the study species showed a sufficiently large safety margin between maximum plant temperature and heat damage threshold. Temperatures in reproductive structures situated 5–10 cm aboveground are usually far below the heat tolerance thresholds. Targeted in situ measurements in our study and in earlier studies (Larcher and Wagner 1983, 2010) demonstrated that caulescent flowers/inflorescences rarely warm up to >35 °C because they are constantly moving due to air convection. Remarkably, in closed alpine grassland, caulescent flowers heat up more than the leaf canopy, as shown by Dietrich and Körner (2014) in a field survey on 43 species. In their study, at high solar radiation the difference reached up to 12 K in some species, with the highest measured absolute temperature being 39 °C in compound flowers of *Aster alpinus*; in cushion plants, petals of sessile open flowers were found to be slightly cooler than the densely packed leaf canopy. These results are in accordance with our observations in *Silene acaulis*. Galen (2006) demonstrated that under warm conditions transpirational cooling by the perianth reduces the excess of heat, which might reduce the risk of thermal damage. However, this mechanism apparently does not hold for deep-seated floral structures, as ovaries experience the same overheating as the cushion foliage and thus are at risk of being heat damaged.

In summers characterized by regular precipitation and the absence of long periods of excessive heat, all of the plant species investigated here seem to be well adapted to the thermal situation in their environment. Generally, longer and warmer seasons are beneficial for reproductive output (e.g. Kudo 1991; Molau 1997, Wagner and Reichegger 1997; Arft et al. 1999; Ladinig and Wagner 2005; Klady et al. 2011; Ertl 2013). However, excessive heat, as occurred in 2003, can cause substantial reproductive losses, particularly in lower alpine belts and in plants on shallow soil (Jolly et al. 2005; Ladinig and Wagner 2005, 2007; Abeli et al. 2012a; Wagner et al. 2012). Plants may recover when regular summers follow, and a reduced reproductive performance during heat waves may even be overcompensated for in the following year as resources are saved by non-flowering plants (Abeli et al. 2012b). The situation will be different when hot and dry summers become more frequent, which is expected as a consequence of rising global temperatures. The greatest temperature increase is anticipated in mountain systems and in the arctic/subarctic (Beniston et al.^1997^; Saetersdal and Birks 1997; Arft et al. 1999; Theurillat and Guisan 2001; Beniston 2003; Nogués-Bravo et al. 2007). The warming effect puts particular pressure on plant populations at the margins of their ecological ranges (Lesica and McCune 2004; Abeli et al. 2012b). Although elevated temperatures do not necessarily cause heat damage, excessive warmth can negatively affect physiological processes and, subsequently, productivity and reproductive performance in plants from cold ecosystems (Marchand et al. 2006; Orsenigo et al. 2014 for review). As a consequence, thermophilous species may replace the less competitive and slow-growing cryophilic species of open habitats, as already evidenced for several summit sites in European mountain systems (Gottfried et al. 2012; Dullinger et al. 2012). Similarly, simulation studies on climate warming in open-top chambers in mountain areas have shown that less competitive species will not benefit from an increase in temperatures (Stenström et al. 1997; Totland and Alatalo 2002; Kudo and Suzuki 2003; Liu et al. 2012). In addition to the competitive pressure caused by differences in growth strength and height, a heat-related decrease in reproductive output in heat-susceptible species could enhance the process of displacement.

Heat effects on the reproductive performance of alpine and arctic species are poorly investigated. Among the reproductive functions, floral induction is particularly susceptible to developmental disturbances. Higher temperatures are known to cause imbalances in signaling and the regulation of resource allocation, which may promote vegetative growth to the disadvantage of generative development (Ruan et al. 2010). In most alpine plants, floral initiation starts in the season before anthesis (Körner 2003; Wagner et al. 2012, and citations therein). Environmental conditions at that time have a significant influence on the number of shoots becoming floral and giving rise to inflorescences in the following year. Warm temperatures may remove the vernalization effect, as has been observed in several mountain species on the Tibetan Plateau (Liu et al. 2012) and for *S. caesia* (A. Seiwald and J. Wagner, unpublished results). Abeli et al. (2012b) conducted a long-term study on the inflorescence production of four alpine plant species and found an individualistic response to temperature. Depending on the species, more summer warmth either stimulated floral induction or led to a decline in inflorescence formation. Equally prone to malfunction are meiosis, pollen-tube growth and pistil functions, which are already disturbed by exposure to 30 °C for few hours (for mountain plants, see Steinacher and Wagner 2012). Generally, excessive heat waves negatively affect fruit set in alpine plants (Orsenigo et al. 2014), particularly in combination with drought (Giménez-Benavides et al. 2007). Reproduction in a broader sense also includes germination and seedling growth—two further developmental stages which are most vulnerable to climatic extremes. On average, seedling mortality rates of alpine plant species are within the rates for a wide range of perennial species (Forbis 2003; Forbis and Doak 2004); however, they drastically increase at stress-dominated bare ground sites (Niederfriniger and Erschbamer 2000). There, in addition to frost (Marcante et al. 2012), heat and drought are major threats to seedling growth (Marcante et al. 2014).

It can be assumed that the individualistic response of species to heat will alter the population dynamics and thereby the composition of plant communities. Species with low rates of recruitment, due to an increased frequency of extreme weather events, will be disadvantaged compared to more heat-tolerant species with a higher reproductive capacity (Abeli et al. 2014). To better understand the impact of rising temperatures on the reproduction of different high-mountain species, it is not only important to know the temperatures at which reproductive tissues are killed, but also the high temperature thresholds for successful reproductive functioning.

#### **Author contribution statement**

UL planned, conceived and performed most of the experiments, analysed the data and wrote parts of the manuscript; MP performed infrared video thermography, IB carried out pollen germination tests, SZ contributed substantially to the assessment of heat tolerance, GN contributed to the concept and provided editorial advice, JW contributed to the concept and wrote the final version of the manuscript.

## Electronic supplementary material

Below is the link to the electronic supplementary material.

Supplementary material 1 (PDF 39 kb)
