# Supplementary material for: Is sexual reproduction of high-mountain plants endangered by heat?
Source: Oecologia. 2015 Feb 20;177(4):1195–210. doi: 10.1007/s00442-015-3247-0 (PMC4363489; doi:10.1007/s00442-015-3247-0)
Supplement: Supplementary file 1 — Supplementary material 1 (PDF 39 kb) [file 442_2015_3247_MOESM1_ESM.pdf]

Is sexual reproduction of high-mountain plants endangered by heat?

Ursula Ladinig, Manuel Pramsohler, Ines Bauer, Sonja Zimmermann, Gilbert Neuner, Johanna Wagner

Institute of Botany, Faculty of Biology, University of Innsbruck,

Sternwartestraße 15, A 6020 Innsbruck, Austria

Corresponding author: johanna.wagner@uibk.ac.at

Table S1. Heat tolerance (LT_{50}) of whole vegetative and reproductive shoots and individual reproductive structures of the investigated alpine plant species at different stages of reproductive development (bud stages *b1*, *b2*; *a* anthesis; *f* early fruit development).

		Visual assessment of whole shoots		TTC-assessment of reproductive structures				
		vegetative	reproductive	pedicel	petals	ovary	style	pollen
Calluna vulgaris	*b2 ns*	50.1 ± 1.0 A	49.1 ± 1.7 A	51.5 ± 1.7 a	-	51.6 ± 2.0 a	49.2 ± 2.8 a A	-
	a ns	50.5 ± 0.9 AB	49.6 ± 1.3 A	-	-	-	49.8 ± 2.2 A	-
	*f ***	51.3 ± 0.9 B	48.9 ± 1.3 A	-	-	-	-	-
Loiseleuria procumbens	*b2 ****	49.7± 0.7 A	48.1± 0.9 A	48.5 ± 0.8 a A	48.0 ± 0.0 a A	47.8 ± 0.8 a A	47.8 ± 0.8 a A	51.4 ± 1.7 b
	*a ****	51.1 ± 0.5 B	48.1 ± 0.7 A	49.1 ± 1.3 a A	48.9 ± 1.0 a B	48.6 ± 1.6 a A	48.8 ± 1.5 a A	-
	*f ***	52.3 ± 0.9 C	49.9 ± 1.0 B	50.5 ± 1.4 a A	-	49.4 ± 1.3 a B	-	-
Rhododendron ferrugineum	*b1 ****	47.7 ± 0.5 A	44.0 ± 1.5 A	48.6 ± 1.3 b A	44.8 ± 0.4 a A	47.8 ± 2.9 b A	48.3 ± 2.1 b B	50.1 ± 1.2 b A
	*b2 ****	49.5 ± 0.8 B	44.9 ± 1.3 AB	48.1 ± 2.0 bc A	44.8 ± 0.4 a A	46.8 ± 1.8 ab A	47.0 ± 1.6 ab A	50.0 ± 1.1 c A
	*a ****	50.2 ± 0.6 B	43.6 ± 1.3 A	48.6 ± 0.8 bc A	44.8 ± 1.6 a A	47.1 ± 1.7 b A	49.8 ± 1.0 c C	*53.0*
	*f **	49.3 ± 0.6 B	46.6 ± 1.1 B	-	-	46.8 ± 1.8 A	-	
Saxifraga bryoides	*b1 ****	49.7 ± 0.8 A	46.6 ± 1.3 A	-	46.4 ± 1.0 a B	48.2 ± 4.7 a A	48.2 ± 4.7 a A	47.8 ±4.1 a A
	*b2 ****	50.0 ± 0.7 A	45.9 ± 1.4 A	46.5 ± 2.6 a A	45.2 ± 0.4 a A	45.8± 2.3 a B	47.5 ± 2.6 a A	46.9 ±1.8 a A
	*a ****	49.6 ± 0.5 A	46.3 ± 1.5 A	49.4 ±1.0 b B	45.7 ± 1.3 a AB	48.4 ± 1.6 b A	48.0 ± 1.0 b A	
	*f ****	50.0 ± 1.6 A	47.0 ± 2.1 A	48.7 ± 1.5 a B	-	47.8 ± 2.4 a A	-	
Saxifraga caesia	*b1 ***	48.7 ± 0.4 A	47.9 ± 1.0 B	-	-	47.3± 1.8 a B	-	49.4 ± 1.5 b A
	*b2 ****	48.9 ± 0.9 AB	45.5 ± 1.6 A	45.2 ± 2.0 ab A	44.2 ± 1.0 a A	45.4± 2.1 ab A	45.6 ± 1.0 ab A	48.1 ± 5.0 b A
	*a ****	49.7 ± 0.5 B	45.1 ± 1.7 A	45.6 ± 1.9 ab A	45.4 ± 1.8 a A	47.3± 1.2 bc B	47.3 ± 0.9 bc B	*58.8*
	*f ****	49.0 ± 1.2 AB	45.9 ± 1.8 A	47.0 ± 2.8 a A	-	48.3 ± 1.9 a B	-	-
Saxifraga moschata	*b1 ***	48.3 ± 0.9 A	47.2 ± 1.4 A	-	47.3 ± 1.5 a B	47.6 ±1.2 a A	-	48.2 ± 3.2 a A
	*b2 ***	48.5 ± 1.2 A	47.3 ± 1.3 A	47.9 ± 1.1 a A	47.4 ± 0.8 a B	47.8 ± 0.9 a A	47.8 ± 1.0a A	50.3 ± 2.3 b A
	*a ***	48.7 ± 1.1 A	46.0 ± 2.6 B	48.3 ± 1.9 b A	44.3 ± 2.7 a A	48.6 ± 1.5 b A	47.3 ± 1.7 b A	*75.0*
	f ns	49.1 ± 0.8 A	48.0 ± 1.8 A	48.5 ± 0.5 a A	-	47.9 ± 2.0 a A	-	
Saxifraga oppositifolia	*b2*	> 52.0	50.8 ± 2.7 A	52.5 ± 2.2 a B	52.2 ± 2.3 a B	51.8 ± 2.3 a A	51.7 ± 2.5 a A	54.5 ±1.4 a A
	a	> 52.0	48.8 ± 2.4 A	49.0 ± 2.7 a A	48.8 ± 2.8 a A	49.6 ± 2.8 a A	50.2 ± 4.0 a A	> 55.0
	f	> 52.0	51.0 ± 0.0 A	52.1 ± 1.8 a B		51.4 ± 1.8 a A		-
Silene acaulis	*b1****	52.2 ± 0.1 B	49.5 ± 2.4 A	-	48.5 ± 3.5 a A	48.3 ± 3.2 a A	48.2 ± 2.9 a A	48.5 ± 3.0 a A
	*b2 ****	50.9 ± 1.2 A	48.4± 2.7 A	49.3 ± 1.6 ab A	46.2 ± 2.0 a A	49.4 ± 2.8 ab A	48.5 ± 1.5 ab A	50.6 ± 2.3 A
	*a ****	50.9 ± 1.4 A	47.9 ± 2.3 A	49.3 ± 1.9 a A	48.0 ± 1.2 a A	49.9 ± 2.0 a A	49.0 ± 2.1 a A	-
	*f ****	51.0 ± 0.0 A	48.9 ± 1.3 A	48.3 ± 0.9 a A	-	49.8 ± 1.7 b A	-	
Cerastium uniflorum	*b2 ****	49.1 ± 0.5 A	45.6 ± 0.3 A	47.7 ± 1.6 b A	45.8 ± 3.0 a A	47.9 ± 2.4 b A	46.6 ± 2.1 a A	43.9 ± 1.3 a
	*a ****	48.9 ± 1.0 A	45.2 ± 0.4 A	48.6 ± 1.8 b A	45.1 ± 2.3 a A	48.2± 1.5 b A	46.4 ± 2.6 a A	*45.0*
	f ns	49.5 ± 0.5 A	48.4 ± 0.2 B	48.6 ± 1.7 a A	-	48.5 ± 2.3 a A	-	-
Ranunculus glacialis	*b2 **	45.9 ± 0.3 B	45.0 ± 1.4 A	46.0 ± 1.4 a A	45.5 ± 1.1 a A	46.3 ± 2.4 a A	45.7 ± 2.1 a A	44.7 ± 1.2 a
	*a ****	46.6 ± 0.4 C	45.6 ± 1.0 A	46.0 ± 0.0 a A	46.1 ± 0.9 a A	45.7 ± 0.5 a A	45.6 ± 0.7 a A	*50.0*
	f ns	45.0 ± 0.8 A	44.9 ± 1.1 A	45.2 ± 1.6 a A	-	45.1 ± 1.4 a A	-	-

Significant differences between vegetative and reproductive shoots within the same developmental stage are indicated by * ($P \leq 0,05$), ** ($P \leq 0,01$), *** ($P \leq 0,001$), *ns* – not significant; *t*-test. Different capital letters indicate significant differences ($P \leq 0.05$) within whole shoots or the same reproductive structure of a species during different stages of reproductive development, different lower case letters indicate significant differences among mean values of different reproductive structures within a species during the same developmental stage; one-way ANOVA. - not determined or not assignable. LT_{50} values for mature pollen (stage *a*) in italics refer to germinability

Table S2. Heat tolerance (LT_{10}) of whole vegetative and reproductive shoots and individual reproductive structures of the investigated alpine plant species at different stages of reproductive development (bud stages *b1*, *b2*; *a* anthesis; *f* early fruit development).

		Visual assessment of whole shoots		TTC-assessment of reproductive structures				
		vegetative	reproductive	pedicel	petals	ovary	style	pollen
Calluna vulgaris	*b2 ns*	48.5 ± 1.1 A	47.3 ± 1.0 A	50.6 ± 1.6 a	-	50.4 ± 1.8 a	48.4 ± 2.8 a A	-
	a ns	49.6 ± 0.7 A	48.3 ± 1.8 A	-	-	-	48.9 ± 1.9 A	-
	*f **	49.3 ± 1.0 A	48.2 ± 1.0 A	-	-	-	-	-
Loiseleuria procumbens	*b2 **	47.6 ± 1.0 A	47.3 ± 1.0 A	46.7 ± 1.4 b A	46.1 ± 0.0 b A	45.7 ± 1.0 b A	45.8 ± 1.1 b A	49.4 ± 1.3 a
	*a ****	49.2 ± 1.1 B	46.1 ± 1.5 B	48.1 ± 1.9 a AB	47.4 ± 1.9 a A	47.5 ± 2.3 a A	47.7 ± 2.2 a A	-
	f ns	50.6 ± 1.8 B	48.4 ± 2.3 AB	48.7 ± 1.4 a B	-	46.6 ± 2.3 a A	-	-
Rhododendron ferrugineum	*b1 ***	45.4 ± 1.8 A	43.6 ±1.3 AB	46.4 ± 1.2 b A	42.7 ± 0.1 c A	45.8 ± 3.5 abc A	45.2 ± 2.6 b A	48.3 ± 1.9 a A
	*b2****	47.9 ± 1.0 AB	44.4 ± 1.3 AB	45.4 ± 2.9 b A	42.7 ± 0.1 c A	44.7 ± 1.8 bc A	45.0 ± 1.7 b A	48.1 ± 1.8 a A
	a ***	48.6 ± 1.6 B	42.8 ± 1.3 A	46.2 ± 0.2 a A	43.3 ± 1.8 b A	44.5 ± 1.4 b A	47.7 ± 1.8 a B	-
	*f **	48.2 ± 0.9 B	45.2 ± 1.5 B	-	-	43.2 ± 2.6 A	-	-
Saxifraga bryoides	*b1****	48.9 ± 1.0 AB	46.0 ± 1.8 A	-	43.8 ± 1.6 a A	44.8 ± 5.2 a A	44.8 ± 5.3 a A	44.4 ± 5.3 a A
	*b2****	49.3 ± 0.8 B	45.4 ± 0.8 AB	44.9 ± 1.9 a A	44.1 ± 0.0 a A	44.4 ± 1.7 a A	45.7 ± 2.2 a A	44.8 ± 1.7 a A
	*a****	48.2 ± 0.4 A	44.9 ± 1.4 B	48.2 ± 1.2 a B	44.6 ± 1.3 b A	47.0 ± 2.0 a A	46.3 ± 1.5 b A	-
	*f ****	48.6 ± 1.8 AB	46.0 ± 1.3 A	47.4 ± 2.0 a B	-	45.4 ± 3.8 a A	-	-
Saxifraga caesia	*b1 ns*	47.1 ± 0.4 A	47.1 ± 0.9 A	-	-	45.3 ± 1.2 b A	-	48.7 ± 1.7 a A
	*b2 ****	47.2 ± 0.9 A	44.7 ± 0.9 B	44.6 ± 2.4 a A	43.7± 0.8 a A	43.1 ± 1.5 a B	44.0 ± 1.4 a A	46.0 ± 4.8 a A
	*a ****	48.7 ± 0.7 B	44.4 ± 1.7 B	44.6 ± 2.4 a A	44.3 ± 2.2 a A	45.9 ± 1.5 a A	45.8 ± 1.4 a B	-
	*f ***	47.6 ± 1.7 A	45.5 ± 2.0 AB	46.6 ± 3.1 a A	-	47.1 ± 3.2 a A	-	-
Saxifraga moschata	*b1 **	47.5 ± 1.0 A	46.3 ± 1.8 A	-	45.2 ± 2.3 a A	45.7 ± 1.0 a A	-	46.2 ± 4.6 a A
	*b2 ***	47.5 ± 1.1 A	46.8 ± 1.3 A	47.1 ± 1.5 b A	46.4 ± 1.2 b A	46.7 ± 1.4 b A	47.0 ± 1.5 b A	49.8 ± 2.5 a B
	*a ****	47.9 ± 0.9 A	45.0 ± 2.1 B	46.8 ± 2.6 a A	43.9 ± 2.6 b A	47.1 ± 2.0 a A	46.4 ± 1.9 a A	-
	*f ****	47.9 ± 1.2 A	45.8 ± 1.1 AB	46.6 ± 0.3 a A	-	46.4 ± 2.3 a A	-	-
Saxifraga oppositifolia	*b2*	-	49.7 ± 2.7 AB	50.3 ± 2.7 a A	50.1 ± 2.6 a A	48.9 ± 3.8 a A	49.2 ± 3.2 a A	51.2 ± 2.5 a A
	a	-	47.6 ± 1.7 A	47.5 ± 2.8 b B	47.2 ± 2.4 b B	48.3 ± 2.8 b A	48.3 ± 3.3 b A	52.0 ± 0.0 a A
	f	-	50.9 ± 0.0 B	50.1 ± 1.5 a AB	-	49.4 ± 1.0 a A	-	-
Silene acaulis	*b1****	50.3 ± 0.2 A	48.0.± 3.0 A	-	46.9 ± 4.0 a A	46.8 ± 3.0 a A	46.5 ± 2.9 a A	46.1 ± 3.8 a A
	*b2 ***	49.7 ± 0.6 B	47.6.± 2.6 A	48.4 ± 1.9 ab A	44.6 ± 2.2 c A	47.6 ± 2.7 ab A	46.9 ± 1.9 b A	48.4 ± 2.2 a A
	*a ****	49.4 ± 1.2 B	46.5 ± 2.2 B	47.9 ± 1.5 a A	46.3 ± 1.4 b A	48.2 ± 2.5 a A	47.4 ± 1.7 ab A	-
	*f ****	50.3 ± 0.0 A	47.0 ± 1.6 AB	47.2 ± 0.8 a A	-	48.9 ± 2.1 b A	-	-
Cerastium uniflorum	*b2****	48.4 ± 0.7 A	43.3 ± 0.4 A	45.8 ± 1.7 a A	43.8 ± 3.0 a A	45.4 ± 2.7 a A	44.2 ± 2.5 a A	42.8 ± 0.8 a
	*a ****	47.7 ± 0.9 A	43.9 ± 2.3 A	46.2 ± 2.7 a A	43.7 ± 2.2 a A	45.5 ± 2.1 a A	44.5 ± 2.8 a A	
	*f **	48:1 ± 0.1 A	46.5 ± 1.3 A	46.7 ± 3.2 a A	-	46.5 ± 4.0 a A	-	
Ranunculus glacialis	*b2 ns*	44.3 ± 0.5 B	44.4 ± 1.1 A	44.8 ± 1.7 c A	44.9 ± 0.9 c A	42.0 ± 2.3 b A	42.5 ± 2.1 b A	39.1 ± 1.2 a
	*a ****	46.2 ± 0.4 C	44.9 ± 0.6 B	43.6 ± 0.0 a A	45.7 ± 0.9 b B	43.8 ± 1.0 a A	43.9 ±1.4 a B	-
	*f ***	43.7 ± 0.8 A	44.2 ± 1.2 A	43.9 ± 1.6 a A	-	43.7 ± 0.8 a	-	-

Significant differences between vegetative and reproductive shoots within the same developmental stage are indicated by * ($P \leq 0{,}05$), ** ($P \leq 0{,}01$), *** ($P \leq 0{,}001$), *ns* – not significant; *t*-test. Different capital letters indicate significant differences ($P \leq 0.05$) within whole shoots or the same reproductive structure of a species during different stages of reproductive development, different lower case letters indicate significant differences among mean values of different reproductive structures within a species during the same developmental stage; one-way ANOVA; - not determined or not assignable

Table S3. Results of a two-way fixed factor analysis of variance (GLM) of the effects “species” and “stage” (bud stage b1, b2, anthesis, fruiting), and their interaction on heat tolerance (LT_{50}) of vegetative shoots. DF degrees of freedom, F variance ratio, P error probability.

Source of variation	DF	F	P
species	8	129.656	> 0.001
stage	3	6.423	> 0.001
species * stage	20	5.741	> 0.001
model	31	38.149	> 0.001
error	339		
R^2 = 0.777			

Table S4. Results of a three-way fixed factor analysis of variance (GLM) of the effects “species”, “stage” (bud stage *b1*, *b2*, anthesis, fruiting) and reproductive “structure” (pedicel, petals, style, ovary), and their interactions on heat tolerance (LT_{50}) of reproductive shoots. DF degrees of freedom, F variance ratio, P error probability.

Source of variation	DF	F	P
species	9	67.5	> 0.001
stage	3	3.5	0.015
structure	3	21.7	> 0.001
species * stage	21	5.1	> 0.001
species * structure	26	3.3	> 0.001
stage * structure	7	1.3	0.269
species * stage * structure	36	1.2	0.172
model	105	10.0	> 0.001
error	1082		
R^2 = 0.494			
